# Impact of treatment on resting cerebral blood flow and metabolism in obsessive compulsive disorder: a meta-analysis

**DOI:** 10.1038/s41598-017-17593-7

**Published:** 2017-12-12

**Authors:** A. L. van der Straten, D. Denys, G. A. van Wingen

**Affiliations:** 1 Department of Psychiatry, Academic Medical Center, University of Amsterdam, Amsterdam, The Netherlands; 2 Brain Imaging Center, Academic Medical Center, University of Amsterdam, Amsterdam, The Netherlands; 3 Amsterdam Neuroscience, Academic Medical Center, University of Amsterdam, Amsterdam, The Netherlands; 4 Amsterdam Brain and Cognition, University of Amsterdam, Amsterdam, The Netherlands

## Abstract

Neurobiological models of obsessive-compulsive disorder (OCD) posit that its clinical symptoms such as repetitive thoughts and behaviors are related to hyperactivity in the cortico–striato–thalamo–cortical (CSTC) circuit. Small scale neuroimaging studies have shown that treatment of OCD is associated with reduced activity across different brain structures within this circuitry. We performed the first meta-analysis of positron emission tomography (PET) and single photon emission computed tomography (SPECT) studies that investigated cerebral blood flow or glucose metabolism in patients with OCD before and after pharmacological or psychological treatment. We calculated standardized mean differences for the regions-of-interest most often reported. The meta-analysis revealed small reductions in activity in the caudate nucleus and orbitofrontal cortex after treatment with a serotonin reuptake inhibitor or cognitive behavioral therapy. Small reductions were also observed in the thalamus when one SPECT study with a large opposite effect was excluded from the analysis. Meta-regression analyses for the caudate nucleus showed no significant effect of the type of treatment, decrease in symptom severity, mean duration until the follow-up scan, or year of publication. These results show that pharmacological and psychological treatments reduce resting CSTC circuit activity, and provide further support for the CSTC circuit model in OCD.

## Introduction

Obsessive-compulsive disorder (OCD) is a severe psychiatric disorder that occurs in 2–3% of the general population^1^. OCD is characterized by repetitive thoughts (obsessions) and repetitive behaviors (compulsions). Compulsions such as washing, cleaning or counting occupy the patients for the entire day rendering them incapable of the routine chores of everyday life. Patients with OCD initially receive pharmacological treatment with selective serotonin reuptake inhibitors (SSRIs) or psychological treatment with cognitive-behavioral therapy (CBT). If the patients do not benefit sufficiently from these treatments, the tricyclic antidepressant clomipramine or addition with antipsychotics are prescribed^2^. Symptom reduction, often quantified with the Yale-Brown Obsessive Compulsive Scale (YBOCS), is the primary measure of success in the treatment of OCD^3^. Neuroimaging studies have implicated the cortico–striato–thalamo–cortical (CSTC) circuit in the pathophysiology of OCD. A disbalance between ventral and dorsal CSTC circuits is thought to lead to increased anxiety, repetitive behaviors and the inability to modulate responses^4^. The main aim of functional neuroimaging studies investigating the effects of treatment is assessing changes in brain activity between a pre- and post-treatment scan. Imaging techniques that have been used most often are 18fluorodeoxyglucose positron emission tomography (FDG-PET), single photon emission computed tomography (SPECT) and functional magnetic resonance imaging (fMRI). FDG-PET and SPECT are both measures of neural activity at rest. fMRI typically measures neural responses to emotional and cognitive challenges that vary substantially across studies, which hampers pooling of those studies for meta-analysis. Our analysis will therefore focus on the resting nuclear neuroimaging studies that have been conducted in OCD.

FDG-PET is an imaging technique that measures the local cerebral metabolic rates for glucose (LCMRglc). The most common finding in FDG-PET studies in patients with OCD is decreased LCMRglc in the caudate nucleus after treatment^5–9^. Others found changes in glucose metabolism in the thalamus, anterior cingulate cortex or orbitofrontal cortex^8,10–12^. The finding of increased glucose metabolism in the caudate nucleus after successful treatment is in conflict with the other studies^13^. Technetium-99m hexamethylpropylene-amine-oxime (HMPAO) and xenon (Xe-) SPECT techniques measure the cerebral regional cerebral blood flow (rCBF). Studies using these techniques report changes in the rCBF after treatment in the caudate nucleus, putamen, thalamus, orbitofrontal cortex and prefrontal cortex^14–19^. Results indicating that pharmacotherapy was associated with an increased rCBF in the thalamus differed from these earlier studies^20^.

The majority of studies reviewed above have pointed towards a reduction of activity in at least one of the brain regions that are part of the CSTC circuit. However, the particular brain regions implicated vary across studies, suggesting more heterogeneity of the results than what is immediately apparent. Moreover, the results of those studies are typically based on small sample sizes, limiting the generalizability of the findings from the individual studies. To overcome these limitations, we performed the first meta-analysis of studies that have assessed resting cerebral blood flow or glucose metabolism in patients with OCD before and after pharmacological or psychological treatment. To explore whether variability in the study methods (e.g., type of therapy) and patient characteristics (e.g., level of symptom improvement) could explain variability in the results, we performed additional meta-regressions.

## Methods

### Literature search and study selection

We searched the Cochrane, PubMed and Psych INFO databases between January 1990 and July 2017 using the following search terms: (cognitive behavioral therapy OR serotonin reuptake inhibitor OR sertraline OR fluvoxamine OR clomipramine OR fluoxetine OR paroxetine OR (es)citalopram) AND (positron-emission tomography OR single-photon emission computed tomography OR glucose metabolism OR regional cerebral blood flow) AND obsessive compulsive disorder. We also manually searched reference lists of published review articles. All retrieved studies subsequently underwent a selection process consisting of reading the articles’ methods sections and applying the following inclusion criteria: (i) Diagnosis: participants of all ages with OCD according to DSM, (ii) Treatment: CBT, an SSRI or tricyclic antidepressant, (iii) Imaging technique: FDG-PET measuring the LCMRglc or SPECT measuring the regional cerebral blood flow, (iv) Analysis: region-of-interest (ROI) based and whole brain, hemisphere or cerebellum ratios. Exclusion criteria were based on case reports, whole brain analyses and studies providing insufficient data (i.e. no information on pre- and post-treatment mean and standard deviations to calculate the effect size). The search and selection of articles was performed by AvdS and GvW and any discrepancy was solved by agreement. The inter-rater agreement score kappa was calculated.

### Extracting data

Characteristics of the subjects and trials from the included studies were collected as followed: (a) group size, (b) type of treatment, (c) imaging technique, (d) duration until follow up (e) mean age, (f) pre-treatment YBOCS score, (g) percentage response rate measured with YBOCS (h) Hamilton Depression Rating scale (HDRS) score [Table 1]. In addition, we determined whether the studies included patients with comorbidity, if and how often they scanned healthy controls and how they defined their regions of interests. The authors described 36 regions of interest in total, which were grouped for further analysis [Table 1]. Although a meta-analysis can be performed with only two studies^21^, we chose to perform the analysis with a minimum of eight studies for increased power of the statistical tests.

**Table 1:** Characteristics of PET and SPECT neuroimaging studies and pre-post treatment effect sizes by region.

Authors	N^1^	Treatment	Imaging	Rescan (weeks)	Mean age (years)	YBOCS score	HDRS	YBOCS decrease (%)	Effect size (g)							
									caudatus	OFC	thalamus	ACC^2^	putamen	PFC^3^	hippo-campus	amygdala
Benkelfat *et al*. 1990	8	TCA	PET	12	32.0 ± 6.6	—	9 ± 3	—	−0.13	−0.47	0.11	—	−0.15	—	0.17	—
Baxter *et al*. 1992	6	SSRI	PET	12	31.2 ± 12.9	25 ± 4	10 ± 5	36	−0.35	0.17	−0.62	−0.27	0.25	—	—	—
	5	CBT	PET		34.7 ± 6.0	24 ± 6	8 ± 6	25	−0.41	−0.19	−0.06	−0.11	−0.22	—	—	—
Swedo *et al*. 1992	13	TCA/SSRI	PET	52	27.8 ± 7.5	—	9 ± 9	—	−0.08	−0.68	—	−0.41	—	−0.17	−0.08	—
Hoehn-Saric *et al*. 1992	6	SSRI	SPECT	14	33.3 ± 9,8	24 ± 4	—	47	—	0.12	—	—	—	—	—	—
Rubin *et al*. 1995	8	TCA	SPECT	30	33.6 ± 5.0	21 ± 5	—	48	0.14	—	0.00	—	0.40	—	—	—
Schwartz *et al*. 1996	9	CBT	PET	12	33.4 ± 6.6	26 ± 4	4 ± 3	49	−0.53	—	—	—	—	—	—	—
Saxena *et al*. 1999	20	SSRI	PET	8–12	—	26 ± 6	9 ± 3	38	−0.32	−1.02	−0,64	−0.34	—	—	—	—
Saxena *et al*. 2002	25	SSRI	PET	8–12	37.5 ± 12.6	26 ± 5	10 ± 4	22	−0.28	−0.23	−0.23	−0.20	−0.22	−0.13	0.00	0.00
Nakatani *et al*. 2003	22	CBT	SPECT	32	29.7 ± 12.0	27 ± 5	14 ± 10	55	−0.46	—	−0.16	—	—	−0.24	—	—
Ho Pian *et al*. 2004	15	SSRI	SPECT	12	30.6 ± 11.0	28 ± 4.9	8 ± 4	21	−0.28	0.14	−0.13	—	−0.56	—	—	—
Diler *et al*. 2004	18	SSRI	SPECT	12	13.2 ± 1.6	—	—	—	−0.90	−0.53	—	−0.90	—	−0.80	—	—
Saxena *et al*. 2009	10	CBT	PET	4	46.4 ± 9.9	25 ± 3	12 ± 5	55	0.11	0.00	−0.32	0.00	0.09	0.21	0.18	−0.30
Apostolova *et al.* 2010	7	SSRI	PET	20	30.9 ± 4.6	23 ± 8	—	43	0.00	−0.17	—	—	—	—	—	—
	9	CBT	PET		37.1 ± 10.3	23 ± 8	—	47	0.12	0.09	—	—	—	—	—	—
Karadag *et al*. 2013	7	SSRI	SPECT	33	33.7 ± 9.2	28 ± 4	10 ± 6	54	—	—	1.11	0.48	—	—	—	—

Characteristics of functional neuroimaging studies and pre-post treatment effect sizes for the caudate nucleus, orbitofrontal cortex (OFC), thalamus, anterior cingulate cortex (ACC), putamen, prefrontal cortex (PFC), hippocampus and amygdala. Treatment: tricyclic antidepressant (TCA), selective serotonin reuptake inhibitor (SSRI) or cognitive behavioral therapy (CBT), imaging techniques: positron emission tomography (PET) and single photon emission computed tomography (SPECT), questionnaires: Yale-Brown Obsessive Compulsive Scale (YBOCS), Hamilton Depression Rating scale (HDRS).

^1^N = all patients (responders and non-responders).

^2^Anterior cingulate cortex or ventral anterior cingulate cortex.

^3^Prefrontal cortex or dorsolateral prefrontal cortex.

### Effect size computation

Comprehensive Meta-Analysis software^22^ was used to perform the analysis. All included studies used the mean ± standard deviation of the sample to report their results. We included all the results reported, significant and non-significant. A separate meta-analysis was performed for each region of interest, averaging the results of both hemispheres. We calculated the standardized mean difference because the studies measured the outcome in a variety of ways. For the studies that reported the results of responders and non-responders separately we calculated the weighted average of the effect sizes by summing the means multiplied by the number of responders/non-responders, divided by the total amount of participants. We expressed our results in terms of Hedges g; for small sample sizes it provides a superior estimate of the standardized mean difference^23^. The magnitude of Hedges g can be interpreted using Cohen’s convention as small (0.2), medium (0.5), and large (0.8)^24^. Because we incorporated a group of studies with different sampling frames, we used the random-effects model for the analysis^23^. The results were corrected with the Holm-Bonferroni method for multiple comparisons^25^. To calculate the effect size, pre-post correlations are needed. None of the included studies reported the pre-post correlations. Therefore, we assumed that these correlations were similar to those reported in test retest reliability studies for each imaging technique^26–28^. In the analysis we used the reported correlations of the different regions for FDG-PET studies (0.72–0.88) and SPECT studies (0.54–0.75). For the regions of interest for which we could not obtain any correlation, we chose the most moderate value (r = 0.50).

Cochran’s Q and I^2^ statistic were used to assess statistical heterogeneity^29^. We explored the heterogeneity by performing a sensitivity analyses while excluding the study with pediatric participants and excluding studies with patients with depressive disorder as concomitant Axis 1 diagnoses. Jackknife analysis was also carried out to test the replicability of the results, by repeating the analysis with discarding one different study each time. As described above, we combined the results of two nuclear imaging techniques, in line with previous OCD neuroimaging meta-analyses^30,31^. FDG-PET measures the local cerebral glucose metabolism while SPECT measures the regional cerebral blood flow. These are two different measures and increased glucose metabolism may not always go together with increased blood flow. Therefore, we performed two separate analyses for each imaging technique to determine if combing these results is reasonable. Publication bias was assessed using funnel plots and the Egger regression test^32^. The dataset analyzed during the current study is available from the corresponding author on reasonable request.

### Meta-regression

In addition, we performed a random-effect meta-regression^33^ to determine (1) the differences between treatment with medication and with CBT; (2) the influence of reduction in YBOCS score; (3) the influence of the mean duration until the follow-up scan; (4) the influence of the date of publication. We could only perform meta-regression for the caudate, as meta-regression should not be considered when there are fewer than ten studies in a meta-analysis^34^. The results of the meta-regression were corrected with a Holm-Bonferroni correction for multiple comparisons.

## Results

### Study selection and characteristics

**Figure 1:**
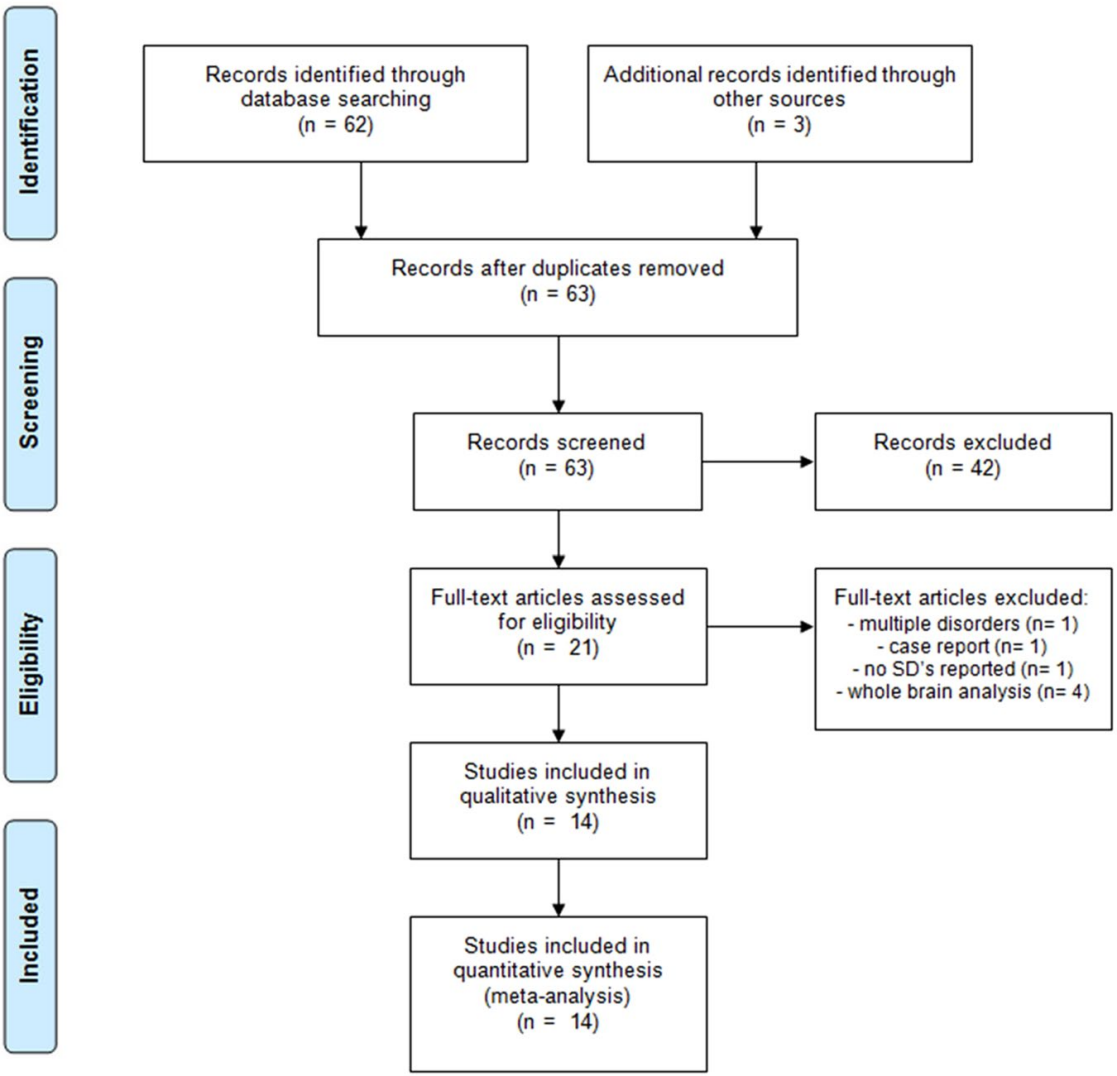
PRISMA flow diagram.

The systematic search retrieved 63 potential articles, of which the title and abstract were scanned for more detailed evaluation. Studies excluded from the analysis were whole brain based^9,14,35,36^ or did not report the standard deviation of the mean^11^. In addition, we excluded studies that investigated a combined group of anxiety disorders^37^ or an individual patient^38^. The flow of studies reviewed is presented in the PRISMA diagram, see Fig. 1 ^39^. After screening with the inclusion criteria, 14 studies that remained were included in the analysis [Table 1]. The inter-rater agreement score kappa was 1.00 after screening the abstracts and 0.68 after screening the full text. The lower inter-rater reliability was due to inclusion or exclusion of three studies that reported regional blood flow or metabolism expressed as percentage rather than proportion. After agreement to include those studies with rescaling to proportion, kappa increased to 1.00. There were eight PET studies and six SPECT studies included, with a total number of 188 patients. Nine studies treated the OCD patients with an SSRI or clomipramine, three studies treated patients with CBT and two studies treated patients with an SSRI or CBT.

While the majority of the studies investigated adult patients, Diler *et al*. included only pediatric patients, aged 11 to 15^19^. The majority of studies excluded patients with comorbid disorders, although this was not always clearly described^10^. In the other studies patients with depressive disorder^5,13,40^ or other concomitant diagnoses like dysthymic disorder, panic disorder or tics participated^6,8,12,17^. Four studies scanned healthy controls before and after treatment^6,12,20,40^, other studies only scanned healthy controls before treatment started^5,15,19^. Because a large proportion of studies did not include data from a healthy comparison group, we performed the analysis without reference to healthy controls. All studies were performed under similar conditions; in a quiet room with as little distractions as possible. During the administration there were no tasks performed. The process of defining the ROIs differed between studies, while some created the ROIs manually for each individual patient on the PET/SPECT scan^6–8,15,18,20^ or on a co-registered structural MRI^12,16,40^, others used a predefined FDG template^13^, a cortical circumferential profiling method^17^ or predefined slices/boxes^5,19^ and made no individual adjustments. Also the rater blindness to subject identity and diagnosis differed between studies.

### Meta-analysis

**Figure 2:**
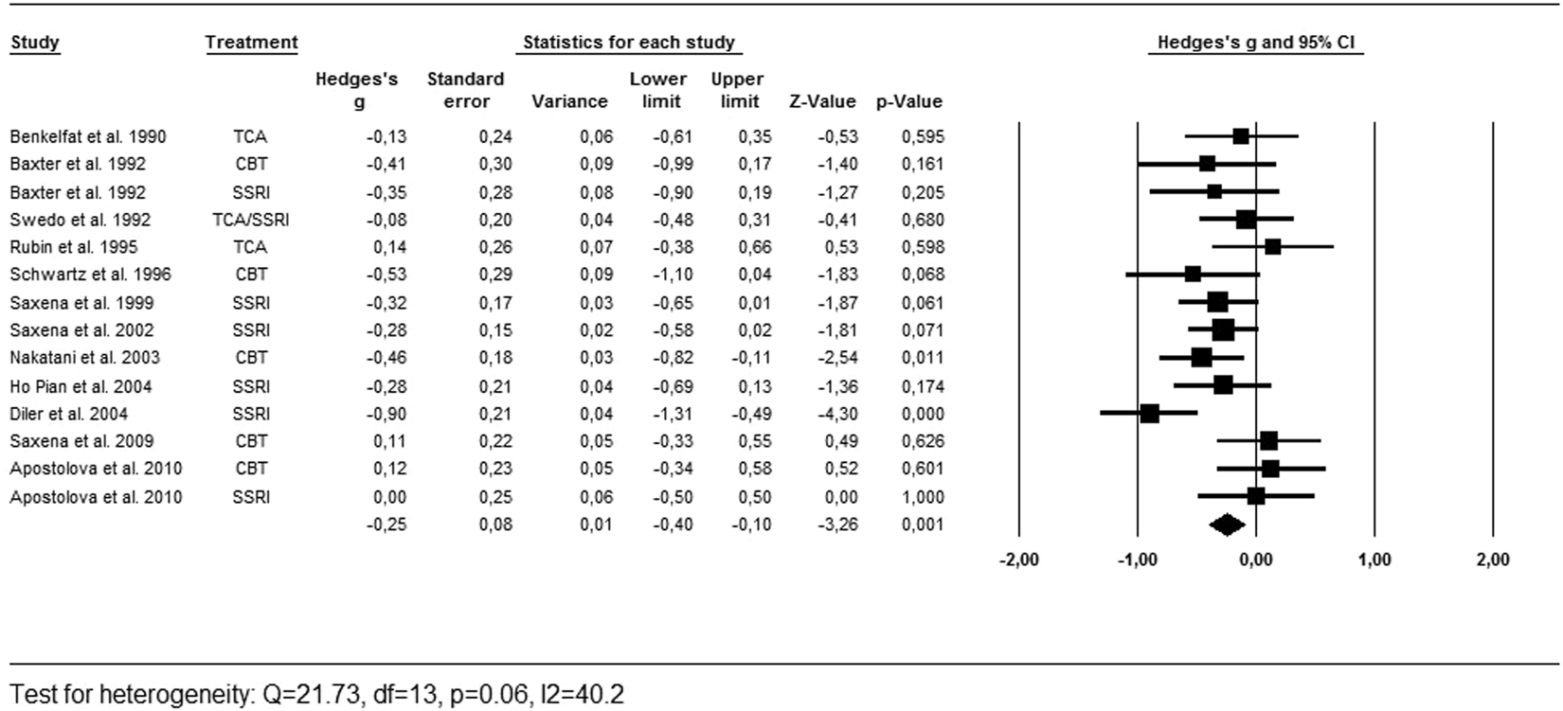
Forest plot of effect sizes of the caudate nucleus for PET and SPECT studies combined.

**Figure 3:**
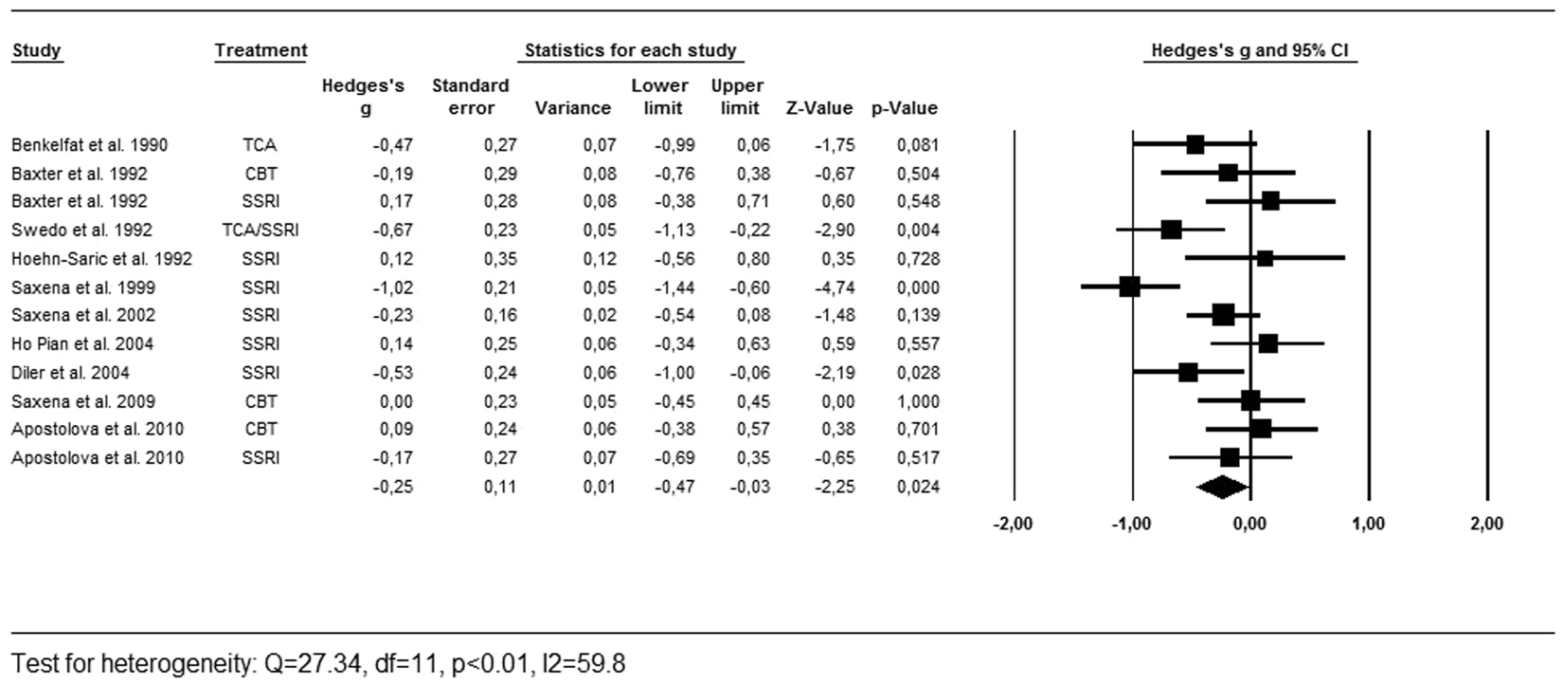
Forest plot of effect sizes of the orbitofrontal cortex for PET and SPECT studies combined.

**Figure 4:**
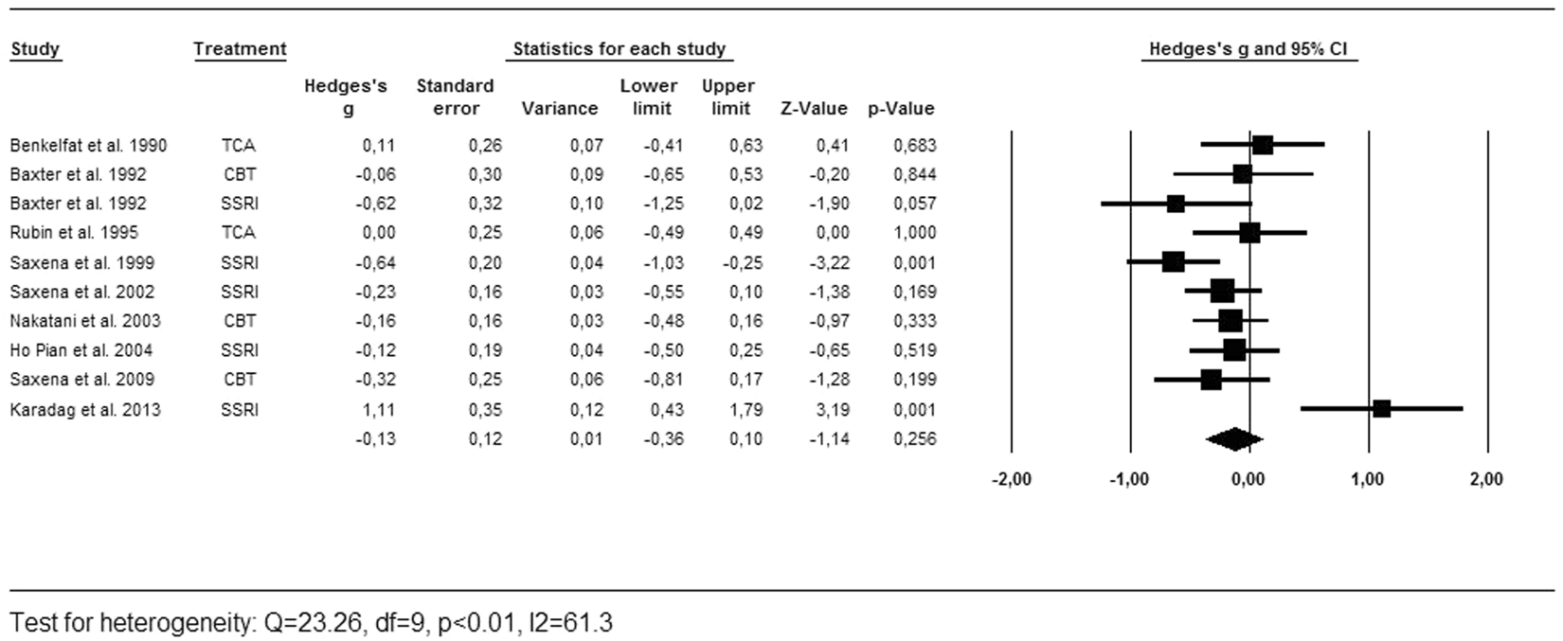
Forest plot of effect sizes of the thalamus for PET and SPECT studies combined.

As described above, we calculated the effects sizes for the regions most often reported by the included studies [Table 1]. Successful treatment led to a significant decrease of activity in the caudate nucleus (g = −0.25, 95% CI = −0.40, −0.10, k = 12, Z = −3.26, p < 0.01, Q = 21.73, df = 13, p = 0.06, I^2^ = 40.2) and the OFC (g = −0.25, 95% CI = −0.47, −0.03, k = 10, Z = −2.25, p = 0.05, Q = 27.34, df = 11, p < 0.01, I^2^ = 59.8). There was no significant effect found in the thalamus (g = −0.13, 95% CI = −0.36, 0.10, k = 9, Z = −1.14, p = 0.26, Q = 23.26, df = 9, p < 0.01, I^2^ = 61.3). As shown in the forest plots [Figs 2, 3 and 4], the average magnitude of the effect sizes found in the analysis were small (0.13–0.25), ranging from small negative effects to large positive effects. The heterogeneity of the results was substantial for the studies looking at the OFC (Q = 27.34, df = 11, p < 0.01, I^2^ = 59.8) and the thalamus (Q = 23.26, df = 9, p < 0.01, I^2^ = 61.3), and was moderate in the caudate nucleus (Q = 21.73, df = 13, p = 0.06, I^2^ = 40.2). We explored heterogeneity by performing sensitivity analyses while excluding studies with aberrant patient characteristics. By removing the studies that included patients with depressive disorder as comorbid diagnosis from the analysis, heterogeneity did not improve substantially. Excluding the study with pediatric participants led to a slight improvement in heterogeneity of the caudate results. The heterogeneity among studies was accounted for by using the random-effects model for the meta-analyses^23^. The jackknife sensitivity analysis on the overall results indicated that the decreased activity in the caudate nucleus was highly replicable, as this finding was preserved throughout all the combinations of studies. The effect sizes became a trend towards significant by repeating the analysis with discarding two of the studies for the OFC^10,19^. After removing one study from the analysis, the effect size for the thalamus became highly significant (g = −0.23, 95% CI = −0.38, −0.07, k = 8, Z = −2.67, p < 0.01, Q = 9.59, df = 7, p = 0.30, I^2^ = 12.5)^20^.

In addition, we performed a separate analysis for each imaging technique, because increased glucose metabolism does not always lead to increased blood flow. The effect directions were negative in the separate analyses for both imaging techniques, indicating a decrease of blood flow and glucose metabolism in all the regions of interest after treatment. FDG-PET studies showed a significant decrease of glucose metabolism in the caudate nucleus (g = −0.18; 95% CI = −0.32, −0.05, k = 8, Z = −2.65, p < 0.01, Q = 7.64, df = 7, p = 0.57, I^2^ = 0.0), the OFC (g = −0.29; 95% CI = −0.54, −0.03, k = 7, Z = −2.23, p = 0.03, Q = 21.87, df = 6, p < 0.01, I^2^ = 63.4) and the thalamus (g = −0.30; 95% CI = −0.53, −0.07, k = 5, Z = −2.61, p < 0.01, Q = 7.13, df = 4, p = 0.21, I^2^ = 29.8). SPECT studies only showed a significant decrease of blood flow in the caudate nucleus (g = −0.40; 95% CI = −0.78, −0.01, k = 4, Z = −2.03, p = 0.04, Q = 10.21, df = 3, p = 0.02, I^2^ = 70.6). There were no signs of publication bias by visually screening the funnel plots of the analyses of the caudate nucleus and OFC. In addition, the Egger’s regression showed no evidence for publication bias for the caudate nucleus (bias = 1.11, 95% CI = −2.79, 5.02, p = 0.54) and the OFC (bias = 1.99, 95% CI = −3.33, 7.31, p = 0.42). The funnel plot and Egger’s regression test for the analysis of the thalamus suggested possible publication bias, with an outlier to the right side (bias = 3.06, 95% CI = −0.46, 6.58, p = 0.08) that had a large influence on the results.

### Meta-regression

As described above, we could only perform a meta-regression for the caudate nucleus as meta-regression should not be considered when there are fewer than ten studies in a meta-analysis. Meta-regression analyses showed no difference between CBT and medication-induced effects on the caudate nucleus. Similarly, there was no significant influence of level of symptom improvement, mean duration until follow up scan and year of publication.

## Discussion

The aim of our meta-analysis was to aggregate the results from previous small sample studies that investigated the impact of treatment on brain activity in OCD. The analysis that combined PET and SPECT imaging studies showed that successful treatment reduced activity in the caudate nucleus and the OFC. There was no significant effect observed in the thalamus, but the jackknife sensitivity analysis showed that this was due to one particular study^20^. By removing this study from the analysis the decrease in activity was highly significant. The average effect sizes were small, presumably due to the combination of studies reporting medium to large effects and studies reporting no effect in the different brain structures. Our results support the hypothesis that symptom reduction is associated with a decrease of resting cerebral blood flow and glucose metabolism in the CSTC circuit. In the separate analyses for these imaging techniques, FDG-PET studies showed a significant decrease of glucose metabolism in the caudate nucleus, the OFC and the thalamus. SPECT studies only showed a significant decrease of blood flow in the caudate nucleus. Meta-regression analysis showed no differences between CBT and medication-induced effects on the caudate nucleus. No significant effects of the reduction in YBOCS score, year of publication and the mean duration until the follow up scan were detected.

The analysis showed normalization of activity in the caudate nucleus, the area that that showed a significant difference in activity between OCD patients and controls in a previous meta-analysis^31^. Our results partially corroborate the conclusions from a recently published systematic review^41^, which reported decreased activity in the ventral CSTC circuits and increased activity in the dorsal CSTC circuits during cognitive processing as assessed with FDG-PET, SPECT and fMRI after psychotherapy in OCD patients. However, the increased activity in the dorsal circuit was only reported in fMRI studies using cognitive paradigms, which were not included in our analysis. And because of the limited amount of studies, we could not perform a meta-analysis on all the regions of interest described in the existing literature, nor perform a meta-analysis on results that have been reported using whole brain analyses (e.g.,^9,14,35^). Remarkable is that the jackknife sensitivity analysis showed that the non-significant result of the thalamus was due to one study with a large positive effect, which is in conflict with the previous literature^20^. A possible explanation of this inconsistent finding might be the fact that they analyzed their scans semi-quantitatively and selected the best slices for thalamic evaluation manually, which might have introduced a bias in the results compared to methods that averaged data for the entire thalamus. Interestingly, this suggests that the effects of treatment on the thalamus may be different across subregions within the thalamus.

The analysis of CBT and medication-related changes at the level of brain areas and circuits provides a better perspective on the pathophysiology of OCD and the response to different treatments. Our results cannot clarify the etiology of OCD, but do support the link between activity in specific brain circuits and reduction of symptoms after successful treatment^42^. The OFC is seen as a crucial integrating link in emotional processing and plays an important role in the consequences of making decisions. The caudate nucleus integrates cortical information, selects and generates routines in response to the specific stimuli. The anterior nucleus of the thalamus plays a role in emotional expression, sending projections to the anterior cingulate cortex^43^. Keeping the function of these areas in mind, normalization of activity in these areas could lead to a reduction of repetitive thoughts, repetitive behaviors and anxiety. It is still unclear whether the changes in brain function lead to symptom reduction or are a consequence of the symptom reduction. Some of the included studies describe a correlation between the reduction in YBOCS score and changes in brain activity^13,40^. Our meta-regression analysis showed no influence of the decrease in YBOCS score. This might be because the response rates between the studies were similar and we performed the meta-regression on study level.

It is a general assumption that increased glucose metabolism leads to increased blood flow, as the blood has to supply the glucose and has to carry away the metabolic waste products. Cerebral glucose metabolism and regional blood flow have been reported to be closely correlated in healthy controls^44^. Altered coupling of cerebral glucose metabolism and regional cerebral blood flow have been reported in major depressive disorder^45,46^. Our results showed no sign of uncoupling between cerebral blood flow and cerebral glucose utilization during recovery, as both of the measures decreased in the analyzed regions.

The findings of our meta-analysis are limited by a number of factors. In most of the studies the participants were re-scanned after 8–12 weeks, but some were rescanned after months or until recovery. Although our meta-regression showed no effect of the main duration until the follow up scan, the clinical symptoms and the brain change continuously and the different results might therefore correlate with the timing of the second scan. Previous research suggested that the caudate changes may occur earlier in the course of treatment, while cortical changes may occur at the end of treatment^6^. In addition, the lack of healthy controls was an important methodological limitation in the analyzed studies. Changes in activity might be the consequence of passed time or previous exposure to the scanning environment if the results are not compared to a healthy control group that is also investigated twice. Because of the limited amount of studies, we performed the analyses without reference to healthy controls. Heterogeneity was substantial for the studies looking at the OFC and thalamus. Clinical and methodological diversity between the studies are likely to contribute to the heterogeneity. Possible explanations are the use of different imaging techniques and the inclusion of participants in different age groups and with comorbid disorders. In addition, the process of defining the ROIs differed substantially between studies. Some of the studies used a predefined template for the ROIs and made no individual alterations, while others manually draw the regions on the functional or the co-registered structural MRI scan. Because the ROIs are generally drawn manually, they vary with devices, raters and research facilities. This variability may lower the reliability and reproducibility of the results and might therefore contribute to the observed heterogeneity.

Although the funnel plots and Egger’s regression tests showed no evidence of publication bias for the majority of the analyses, this field of research is prone to report results that are in line with the previous literature. The studies predefine their regions of interest based on the previous literature, which might also be contaminated by publication bias. Since recruitment of OCD patients is often challenging, many of the individual studies have small sample sizes, which undermines the reliability of the results. Furthermore, the power to detect bias with the Egger’s regression test is low with a small numbers of studies, so we cannot fully exclude the possibility of publication bias. The question whether treatment with CBT or SSRIs in OCD leads to similar or different functional changes in the brain remains to be addressed. Most of the studies found no differences between the treatment modalities^6,13,40^. Important methodological shortcomings in these studies are the small sample sizes and the lack of randomization. Therefore, neuroimaging studies controlled by a randomized treatment intervention are needed to assess whether SSRIs and CBT also have different effects on the brain.

In conclusion, this meta-analysis revealed consistent though small pharmacological and CBT-induced changes in cerebral blood flow and glucose metabolism in the caudate nucleus and OFC in patients with OCD. There was no significant effect found in the thalamus, although the jackknife sensitivity analysis showed that this was due to a large positive effect in one particular study. Therefore, our results support the hypothesis that symptom reduction in OCD is associated with decreased CSTC circuit activity. Remarkable is the small reduction in activity in the particular brain structures, which is a result of studies reporting medium to large effect sizes combined with other studies reporting no significant effect of treatment.
